# Laryngeal reinnervation for unilateral vocal fold paralysis in adults; a systematic review of the literature for the clinician

**DOI:** 10.1007/s00405-025-09737-7

**Published:** 2025-10-15

**Authors:** Stephanie D. Mes, Bas J. Heijnen, Martine H. Hendriksma, Antonius P.M Langeveld, Emilie A.C. Dronkers, Elisabeth V. Sjögren

**Affiliations:** 1 Department of Otolaryngology and Head- and Neck Surgery, Leiden University Medical Center, Leiden, The Netherlands; 2 Department of Otorhinolaryngology, Groene Hart Ziekenhuis, Gouda, The Netherlands; 3 Department of Clinical Research Unit, Dept. of Internal Medicine, Leiden University Medical Center, Leiden, The Netherlands; 4 Department of ENT, Head and Neck Surgery, Leiden University Medical Center, Albinusdreef 2, Postbus, Leiden, 9600, 2300 RC The Netherlands

**Keywords:** Unilateral vocal fold paralysis, Laryngeal reinnervation, Systematic review, Outcome measures

## Abstract

**Purpose:**

Unilateral vocal fold paralysis (UVFP), resulting from recurrent laryngeal nerve injury, significantly impairs phonation and reduces quality of life. Laryngeal reinnervation, including ansa-to-recurrent (ansaNSR) and nerve-muscle pedicle reinnervation combined with arytenoid adduction (NMPR + AA), has gained attention as a durable treatment option. This systematic review aims to evaluate the outcomes of laryngeal reinnervation, identify predictive factors and highlight knowledge gaps relevant to clinical practice.

**Methods:**

A systematic search of PubMed, Embase, Web of Science, the Cochrane Library and Emcare identified 42 studies. Outcome measures included perceptual, acoustic, aerodynamic, videostroboscopic and patient-reported outcomes. The risk of bias was assessed using the Methodological Index for Non-Randomized Studies (MINORS) tool. Weighted mean improvements were calculated for clinically relevant parameters.

**Results:**

We included forty-two studies (*N* = 1 859 patients) in our review. These studies demonstrated high rates of voice improvement, primarily based on data from the ansaNSR (*N* = 1 369) and NMPR + AA (*N* = 278) techniques. Age and duration of denervation emerged as the most influential predictive factor. Younger patients (< 60 years) and those with shorter denervation periods (< 2 years) had better outcomes, although successful results were also observed beyond these thresholds. The predictive value of preoperative laryngeal electromyography (LEMG), etiology of UVFP and clinical presentation remains inconclusive.

**Conclusion:**

Laryngeal reinnervation (ansaNSR and NMPR + AA) is effective for UVFP, offering durable voice improvements with minimal complication rates. Future research should focus on standardizing outcome measurements, clarifying predictive factors and refining patient selection to enhance clinical decision-making.

**Supplementary Information:**

The online version contains supplementary material available at 10.1007/s00405-025-09737-7.

## Introduction

Unilateral vocal fold paralysis (UVFP) is defined as the immobility of one vocal fold resulting from injury to the recurrent laryngeal nerve. This condition disrupts the coordination of vocal fold movements essential for phonation, leading to symptoms such as hoarseness, breathiness, reduced vocal range and vocal fatigue, significantly affecting patients’ quality of life [1, 2].

Surgical treatment options for UVFP include injection augmentation, laryngeal framework surgery (LFS) procedures such as medialization thyroplasty (MT) with or without arytenoid adduction (AA), as well as laryngeal reinnervation and combinations of these approaches [3]. Among these, laryngeal reinnervation has gained increasing popularity in recent years.

Laryngeal reinnervation was first described by Horsley in 1909, through primary recurrent to recurrent non-selective anastomosis (RRNSA) of the severed recurrent laryngeal nerve [4]. The non-selective ansa cervicalis to recurrent laryngeal nerve technique (ansaNSR) was introduced by Crumley and Ibzdebski in 1986, and the first recurrent-to-recurrent non-selective anastomosis with an interposition nerve graft (RRNSANG) by Berendes & Miehlke in 1968, who used a great auricular nerve graft. The hypoglossal non-selective reinnervation (HNSR) was described by Paniello in 2000 [5, 6]. These techniques restore innervation to the paralyzed vocal fold by creating a new neural connection at the main branch of the recurrent nerve. They are classed as non-selective because they continuously and simultaneously stimulate the abductor and adductor laryngeal muscles, thereby improving vocal fold tone, muscle bulk, arytenoid position and enhancing phonatory tension [7]. Another approach, nerve muscle pedicle laryngeal reinnervation (NMPR), first described by Tucker in 1976, involves inserting a donor nerve branch directly into the adductor muscle complex through a thyroid cartilage window. Strictly speaking, this is not a non-selective technique, as it only reinnervates the adductor complex. This procedure is often combined with AA to optimize arytenoid positioning [8].

Laryngeal reinnervation offers long-term voice rehabilitation without requiring implants, and preserves laryngeal structure. Similar to laryngeal framework surgery, it involves a single surgical intervention, unlike conservative treatments such as voice therapy or injection laryngoplasty, which often require repeated sessions. It is particularly suitable for younger patients, as it does not interfere with the developing larynx and avoids the need for repeated adjustments to voice outcomes [9].

**Fig. 1:**
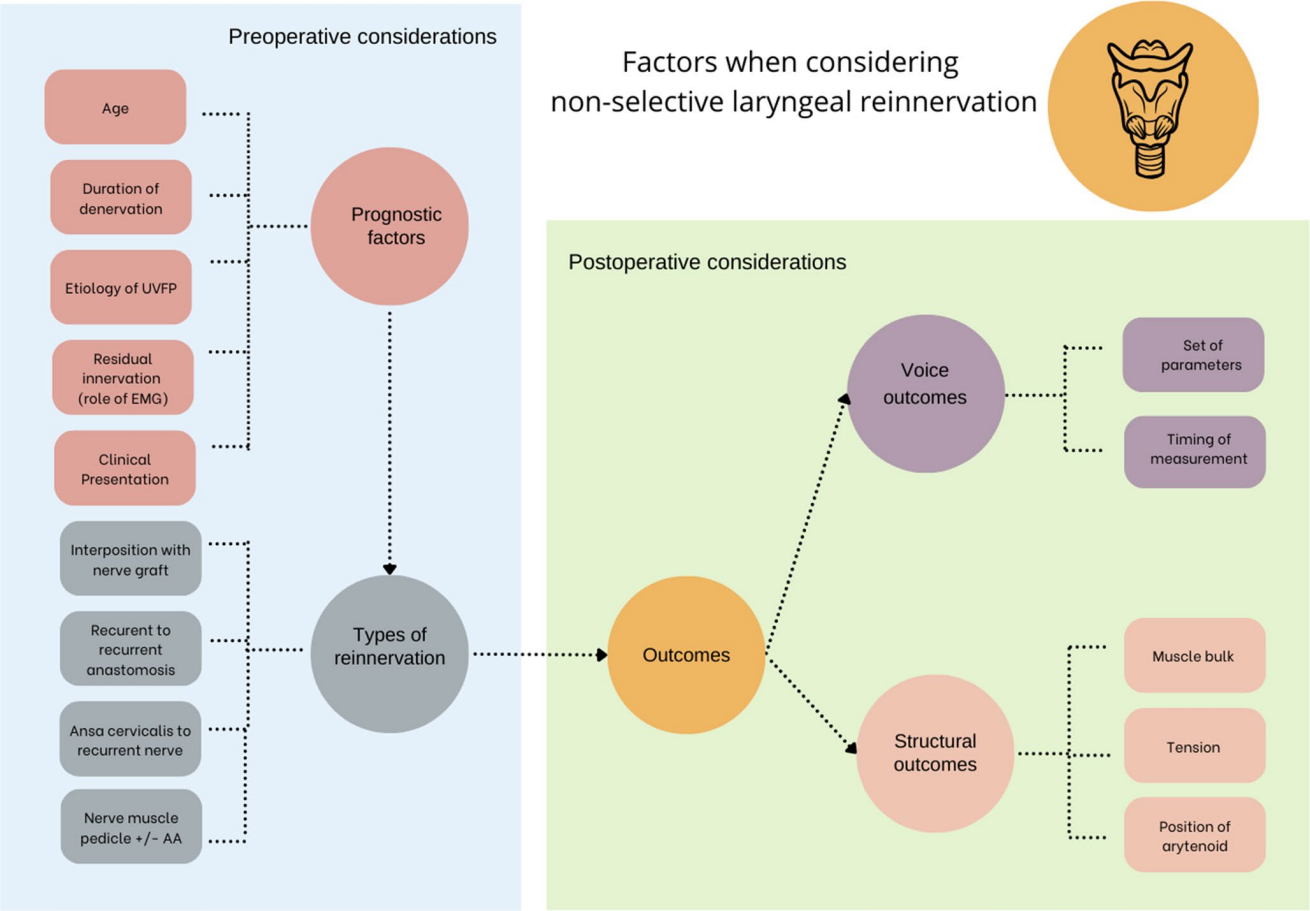
factors when considering non-selective laryngeal reinnervatio

However, selecting the appropriate reinnervation approach requires clinicians to have a sound understanding of the effectiveness and success rates of the different procedures. Insights into postoperative voice outcomes and potential changes in vocal fold configuration—such as tension, muscle bulk and arytenoid position—are essential. Additionally, the influence of predictive preoperative factors must be considered. These include patient age, duration of denervation, paralysis etiology, LEMG findings and clinical presentation (e.g. small vs. large glottal gap, arytenoid position). These parameters are illustrated in Fig. 1. Due to the limited size and heterogeneity of existing data from case series and systematic reviews, preoperative counselling regarding the different treatment modalities remains challenging.

This systematic review aims to:

1. Summarize outcome data for laryngeal reinnervation procedures in adults relevant to clinical counselling;
2. Evaluate the impact of key predictive factors; and
3. Identify gaps in the literature to guide future investigations.

## Methods

### Protocol and registration

This systematic review was conducted in accordance with the Preferred Reporting Items for Systematic Reviews and Meta-Analyses (PRISMA) guidelines [10]. The identified records were uploaded to Rayyan, an online application for independent screening of abstracts [11]. We extracted outcomes of interest from full-text articles and recorded them in Microsoft Excel. The review was registered with the International Prospective Register of Systematic Reviews (PROSPERO) on December 7, 2020 (registration number CRD42021250210).

### Search strategy

We conducted a systematic search on 15 October 2023 using PubMed, Embase, Web of Science, Cochrane Library and Emcare. Additional searches via Google Scholar did not identify other references. The search strategy included a combination of Medical Subject Headings (MeSH) and free-text terms related to unilateral vocal fold paralysis, laryngeal reinnervation, voice quality, surgery and the recurrent laryngeal nerve, along with relevant synonyms (Supplementary material 1). No date restrictions were applied.

### Inclusion and exclusion criteria

Two authors (SDM, BJH) independently reviewed papers based on eligibility criteria, resolving discrepancies through consensus meetings. Full texts were screened for inclusion by the same authors. Included studies reported on non-selective laryngeal reinnervation for UVFP, described the reinnervation techniques and used subjective and objective voice outcome measures. They also had a follow-up time of more than six months for the main techniques. Exclusion criteria included meta-analyses, systematic reviews, non-UVFP pathologies, case studies with fewer than five patients, pediatric populations (< 18 year.), lack of postoperative voice outcomes and unspecified interventions. If multiple articles by the same author appeared to use the same patient cohort, the one with the longest follow-up was included. When clarification was not possible, both studies were included. Additional references were obtained from reference lists of included studies.

### Data extraction

Full-text analyses of included studies focused on demographic data, UVFP etiology, surgical techniques, time since paralysis, objective and subjective voice outcomes and laryngeal electromyography measurements.

### Risk of bias analysis

We assessed the risk of bias using the revised Methodological Index for Non-Randomized Studies (MINORS) [12]. This tool evaluates twelve items, with eight applicable to non-comparative studies and all twelve to comparative studies. Items are scored from 0 (not reported) to 2 (adequately reported), yielding a maximum score of 16 for non-comparative and 24 for comparative studies. Two reviewers (SDM, BJH) independently assessed quality, resolving discrepancies by consensus. The original publication of MINORS does not specify thresholds to categorize studies as high or low quality. A low MINORS score was not a reason for exclusion, to preserve valuable data and to reflect the full scope of available evidence.

### Voice outcome indicators

We assessed the following objective and subjective voice outcome indicators:

- GRBAS scale: Evaluates grade, roughness, breathiness, asthenia and strain, with scores ranging from 0 (normal) to 3 (severe dysphonia) [13]. Studies often calculate mean improvement values [14].
- Jitter/Shimmer: Jitter measures frequency perturbation, while shimmer measures amplitude variability. Normal jitter is < 0.5% and normal shimmer is < 5% [15, 16].
- NHR/HNR: Noise-to-harmonics ratio (NHR) and harmonics-to-noise ratio (HNR) assess noise in voice signals. A lower NHR (< 0.2) and higher HNR indicate better voice quality; normal HNR values are suggested to be 7.4 dB and above [17, 18].
- MPT: Maximum phonation time (MPT) measures the duration of sustained phonation, typically of the vowel/a/. Normal MPT is approximately 20 s for healthy adults [19].
- Videostroboscopy: Evaluates structural outcomes of reinnervation (muscle bulk, tension and arytenoid position) based on glottal closure, mucosal wave, amplitude and regularity. Scores are typically on a scale from 0 (poor) to 3 (good), except in Chhetri et al. (scale 0–5) [20] and Kumai et al. (inverse 0–3 scale) [39].
- Voice Handicap Index (VHI): The VHI-30 and VHI-10 measure voice impairment. The VHI-30 scores range from 0 to 120 across functional, physical and emotional domains; scores < 15 are considered normal and > 60 indicate severe dysphonia. The shorter VHI-10 considers scores > 11 abnormal [21–24].

Subjective analysis primarily relied on VHI scores. Additional measures included subjective voice ratings (on 0–5 or 0–10 scales) and the Voice-Related Quality of Life (V-RQOL) measure. Perceptual evaluation used a 0–7 scale. However, due to limited application and small sample sizes, the analysis focused primarily on VHI for subjective outcomes and GRBAS for perceptual outcomes.

### Predictive factors

The following predictive factors for the outcome of the reinnervation procedure, identified from the literature or personal communications, were selected for review in this study: patient age, resting position of the paralyzed vocal fold, etiology of the paralysis, duration of denervation and the presence of any residual innervation as observed on a preoperative LEMG.

### Statistical analysis

A meta-analysis was not conducted due to substantial heterogeneity in the data across studies. Variations included the etiology of the UVFP, initial vocal fold position, voice outcome indicators, duration of denervation and patient age distribution. These factors prevented valid direct comparisons. We calculated weighted mean improvements for clinically relevant parameters (GRBAS grade, VHI and MPT) per reinnervation procedure.

## Results

### Study selection

**Fig. 2:**
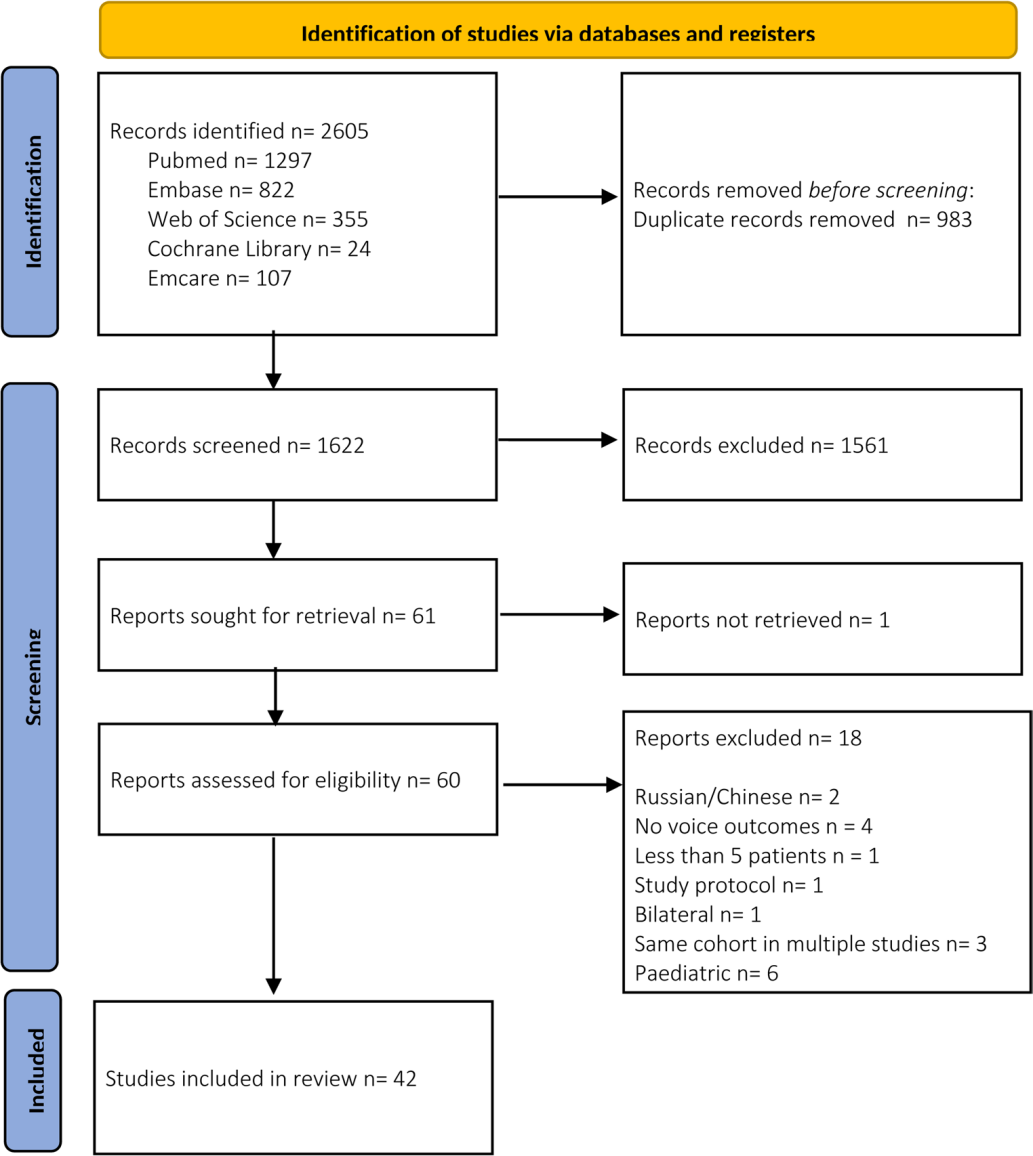
Flow-diagram illustrating searching and selection procedure OR flowchart of search

A flow diagram of the study selection process is presented in Fig. 2. The initial search yielded 2 605 citations, of which 1 622 remained after removal of duplicates. Following title and abstract screening, 1 561 citations were excluded, leaving 61 articles for full-text review. One article could not be retrieved. Eighteen articles were excluded due to non-English language (*n* = 2), pediatric populations (*n* = 6), absence of voice outcome measures (*n* = 4), fewer than five patients (*n* = 1), bilateral procedures (*n* = 1), study protocol descriptions only (*n* = 1), or overlapping cohorts (*n* = 3). Ultimately, 42 studies were included for data extraction and risk of bias assessment, describing a total 1 859 patients who underwent non-selective reinnervation (Supplementary material 2) [5, 20, 25–64].

### Risk of bias analysis

The risk of bias assessment, conducted using the MINORS tool, indicated a moderate risk of bias across studies. Non-comparative studies had a median score of 9.1 out of 18 (range: 4–14) and comparative studies had a median score of 17.2 out of 24 (range: 14–21), reflecting the generally limited methodological quality (Supplementary material 2).

### Study characteristics

Five types of laryngeal reinnervation were identified: ansa cervicalis non-selective reinnervation (ansaNSR), nerve-muscle pedicle reinnervation (NMPR), recurrent to recurrent non-selective anastomosis (RRNSA), recurrent to recurrent non-selective anastomosis with interposition nerve graft (RRNSANG) and Hypoglossal non-selective reinnervation (HNSR). Most cases of NMPR were combined with arytenoid adduction (NMPR + AA, Table 1). We summarized the etiology of UVFP in Table 2, with thyroid cancer or surgery accounting for 43.2% of cases and 35.5% of cases lacking a reported etiology. The mean duration of denervation before surgery was 16.5 months, with 26.5% of patients undergoing preoperative LEMG evaluation. Table 3 outlines the voice outcome parameters used and Tables 4, 5, 6, 7, 8 and 9 summarize the reported voice data.

**Table 1:** Study characteristics

Type of reinnervation	Studies	Number of patients	Mean age
Ansa Cervicalis (ansaNSR)	25	1369	47.7
Nerve Muscle Pedicle (NMPR)	13	278	56.6
Recurrent to recurrent anastomosis (RRNSA)	8	113	53.0
Nerve graft (RRNSANG)	8	90	53.3
Hypoglossal nerve (HNSR)	1	9	57.0

*ansaNSR* ansa cervicalis non-selective reinnervation, *NMPR* nerve-muscle pedicle reinnervation, *RRNSA* recurrent to recurrent non-selective anastomosis, *RRNSANG* recurrent to recurrent non-selective anastomosis with interposition nerve graft, *HNSR* hypoglossal nerve non-selective reinnervation

**Table 2:** Etiology of UVFP

Etiology of UVFP	Total cases	ansaNSR	NMPR	RRNSA	RRSANG	HNSR
Thyroid cancer/surgery	803 (43%)	559	63	92	89	
Not reported	660 (35%)	598	53			9
Iatrogenic not specified	147 (8%)	91	56			
Idiopathic	49 (3%)	44	5			
Aorta aneurysm	30 (2%)	2	28			
Lung cancer	25 (1%)	7	18			
Paraganglioma	16 (1%)	16				
Cervical spine surgery	13 (1%)	13				
Neck dissection	13 (1%)	12	1			
Cerebral surgery	13 (1%)	3	10			
Vagal nerve surgery	10 (1%)	10				
Vagal schwannoma	10 (1%)	1	9			
Aorta surgery	8 (< 1%)	3	5			
Mediastinal tumor	8 (< 1%)	1	7			
cervical trauma	10 (1%)	8			2	
Oesophageal cancer	8 (< 1%)		8			
Skull base surgery	7 (< 1%)	6	1			
Thoracic surgery	6 (< 1%)	3	3			
Thymus tumor	5 (< 1%)		5			
Pneumonectomy	4 (< 1%)	4				
Neck trauma	3 (< 1%)	3				
Tuberculosis	3 (< 1%)	1	2			
Subarachnoid bleeding	3 (< 1%)		3			
Mediastinoscopy	2 (< 1%)	2				
Intubation	2 (< 1%)	1	1			
Wallenburg syndrome	1 (< 1%)	1				
Breast cancer metastasis	1 (< 1%)	1				
Cervix cancer metastasis	1 (< 1%)	1				

*UVFP * unilateral vocal fold paralysis, *ansaNSR * ansa cervicalis non-selective reinnervation, *NMPR * nerve-muscle pedicle reinnervation, *RRNSA * recurrent to recurrent non-selective anastomosis, *RRNSANG * recurrent to recurrent non-selective anastomosis with interposition nerve graft, *HNSR* Hypoglossal non-selective reinnervation

**Table 3:** Voice outcome indicators (VOI)

Voice Outcome indicator	Total	ansaNSR	NMPR	RRNSA	RRNSANG	HNSR
MPT (Maximum phonation Time)	36	16	9	4	6	1
GRBAS scale	29	15	8	3	3	0
Videostroboscopy	20	12	4	2	2	0
Jitter/Shimmer	19	11	5	0	3	0
Laryngeal EMG	15	9	2	1	2	1
MFR (Mean Airflow Rate)	12	1	6	2	3	0
VHI-10/30 (Voice Handicap Index)	10	4	4	1	1	0
HNR (Harmonics/Noise Ratio)	10	2	6	1	1	0
NHR (Noise/Harmonics Ratio)	8	6	1	0	1	0
MPR (Mean Phonatory Resistance)	4	1	3	0	0	0
EAT-10 (Eating Assessment Tool)	3	2	1	0	0	0
V-RQOL (Voice Related Quality of Life	3	1	2	0	0	0
PEI (Phonation Efficiency Index)	3	1	0	1	1	0
Subjective voice 0–5	2	1	0	1	0	0
RSI (Reflux Symptom Index)	1	1	0	0	0	0
Frequency of laryngospasm	1	1	0	0	0	0
Cepstral peak prominence	1	1	0	0	0	0
Rouen Q score	1	1	0	0	0	0
Subjective voice quality 0–10	1	1	0	0	0	0
Maximum intensity	1	1	0	0	0	0
Perceptual score 1–7	1	1	0	0	0	0
Subglottic pressure	1	1	0	0	0	0
Airflow	1	1	0	0	0	0
Resistance	1	1	0	0	0	0
Pitch range	1	0	1	0	0	0
Aspiration 0–3	1	0	0	1	0	0
Voice turbulence index	1	0	0	0	0	1

*ansaNSR* ansa cervicalis non-selective reinnervation, *NMPR * nerve-muscle pedicle reinnervation, *RRNSA * recurrent to recurrent non-selective anastomosis, *RRNSANG * recurrent to recurrent non-selective anastomosis with interposition nerve graft, *HNSR* Hypoglossal non-selective reinnervation

**Table 4:** Perceptual voice analysis – GRBAS scale

GRBAS	Study	Patients (*n*)	G (pre)	G (post)	*R* (pre)	*R* (post)	B (pre)	B (post)	A (pre)	A (post)	S (pre)	S (post)	Follow up time
ansaNSR	Hassan 2012[34]	9	2.20	0.10*			2.00	0.10*					> 24 m
	Li 2014 [42]	349	2.20	0.20*									> 24 m
	Marie 2020 [45]	48	2.15	0.50*	1.34	0.50*	1.63	0.00*	1.39	0.50*	0.20	0.00	36 m
	Wang 2011[56]	237	2.20	0.00*	1.80	0.00*	2.00	0.00*	1.40	0.00*	1.00	0.00*	> 24 m
	Wang 2011[57]	56	2.20	0.20*	1.80	0.20*	2.00	0.00*	1.60	0.00*	1.00	0.00*	> 24 m
	Wang 2020 [58]	37	2.00	0.20*	1.80	0.00*	2.00	0.00*					12 m
	Wang 2020 [59]	13	1.00	0.40	1.00	0.20	0.40	0.00					24 m
	Candelo 2023 [28]	5	3.00	1.00*									18 m
NMPR	Hassan 2011[33]	13	2.20	0.02*			2.20	0.02*					> 24 m
	Hassan 2014 [32]	11	2.40	0.20*			2.00	0.10*					> 24 m
	Kodama 2015 [38]	33	2.50	0.40*			2.40	0.30*					24 m
	Kodama 2017[36]	40	2.50	0.50*			2.50	0.50*					> 12 m
	Sanuki 2015[52]	12	2.50	0.20*	0.10	0.00	2.40	0.20*	0.70	0.10*	0.10	0.00	24 m
	Kodama 2024 [37]	29	2.00	1.00*			2.00	0.50					20 m
	Nishimoto 2023 [48]	68	2.30	0.60*			2.20	0.50					24 m
RRNSA	Chou 2003[29]	8	NR	0.38	NR	0.50	NR	0.25	NR	0.25	NR	0.25	NR
	Rohde 2012[51]	4	NR	1.30	NR	1.30	NR	0.40	NR	0.70	NR	2.40	NR
RRNSANG	Li 2013 [43]	14	2.20	0.60*	1.70	0.40*	1.90	0.00*	1.20	0.00*	1.00	0.00	24 m

*ansaNSR* ansa cervicalis non-selective reinnervation, *NMPR * nerve-muscle pedicle reinnervation, *RRNSA * recurrent to recurrent non-selective anastomosis, *RRNSANG * recurrent to recurrent non-selective anastomosis with interposition nerve graft, *NR * not reported,

*=reported as statistically significant improvement

**Table 5:** Acoustic analysis – HNR/NHR

Jitter/Shimmer	Study	Patients (*n*)	Preoperative jitter (%)	Postoperative jitter (%)	Preoperative shimmer (%)	Postoperative shimmer (%)	Follow up time
ansaNSR	Ab Rani 2021[25]	9	2.24	1.20	8.11	5.97	12 m
	Kumai 2016 [39]	8	NR	1.30	NR	6.00	12 m
	Lee 2018 [40]	19	4.86	0.95*	5.85	2.87*	36 m
	Li 2014 [42]	349	1.82	0.27*	9.49	3.22*	> 24 m
	Marie 2020 [45]	48	13.72	1.33*	1.40	0.40	36 m
	Su 2007 [53]	10	2.19	0.54*	7.18	2.45*	> 24 m
	Wang 2011[56]	237	1.52	0.33*	9.33	4.22*	> 24 m
	Wang 2011[57]	56	1.69	0.52*	10.25	5.37*	> 24 m
	Wang 2020[58]	37	0.93	0.29*	7.03	3.59*	12 m
	Wang 2020[59]	13	0.81	0.71	6.58	6.06	24 m
	Zheng 1996[64]	8	2.03	0.43*	8.83	3.22*	> 12 m
NMPR	Hassan 2014 [32]	11	8.60	1.20*	13.00	4.00*	> 24 m
	Kodama 2017[36]	40	9.00	4.00*	14.00	6.00*	24 m
	Sanuki 2015[52]	12	7.74	0.89*	12.00	5.10*	24 m
	Yumoto 2010 [63]	22	7.67	1.54	12.28	6.04	12–36 m
	Nishimoto 2023 [48]	68	8.90	1.50	14.50	7.70	24 m
RRNSANG	Kumai 2016 [39]	9	NR	0.90	NR	3.90	12 m
	Li 2013[43]	14	1.72	0.30*	8.56	3.58*	24 m
	Yumoto 2006 [62]	8	NR	1.05	NR	2.00	NR

*ansaNSR * ansa cervicalis non-selective reinnervation, *NMPR * nerve-muscle pedicle reinnervation, *RRNSANG * recurrent to recurrent non-selective anastomosis with interposition nerve graft, *NR * not reported,

*=reported as statistically significant improvement

### Perceptual analysis

The GRBAS scale was utilized in 18 studies (total patients, *n* = 986: 754 ansaNSR, 206 NMPR + AA, 12 RRNSA, 14 RRNSANG) [28, 29, 32–34, 36–38, 42, 43, 45, 48, 51, 52, 56–59]. Preoperative grade scores ranged from 1.0 to 3.0 and postoperative scores ranged from 0.0 to 1.3, with improvements ranging from 0.6 to 2.3 points. The weighted mean improvement was − 2.01 for ansaNSR and − 1.82 for NMPR + AA (Table 4).

### Acoustic analysis

Jitter and shimmer were analyzed in 19 studies (total patients, *n* = 978: 794 ansaNSR, 153 NMPR + AA, 31 RRNSANG), with follow-up durations exceeding twelve months [25, 32, 36, 39, 40, 42, 43, 45, 48, 52, 53, 56–59, 62–64]. Jitter improved from 0.81 to 9.00% preoperatively to 0.27–4.00.27.00% postoperatively, while shimmer improved from 1.40 to 14.50% to 0.40–7.70% (Table 6). Noise-to-harmonics ratio and harmonics-to-noise ratio were assessed in 17 studies (total patients, *n* = 947: 729 ansaNSR, 195 NMPR + AA, 1 RRNSA, 22 RRNSANG) [25, 32–34, 36, 37, 40, 42, 43, 48, 52, 56–59, 62, 63]. NHR improved from 0.07 to 0.32 preoperatively to 0.018–0.06 postoperatively, while HNR improved from 3.8 to 17.1 to 7.9–21.8 (Table 7).

**Table 6:** Acoustic analysis – HNR/NHR

Reinnervation type	Study	Patients	Type	Preoperative mean(SD)	Postoperative mean (SD)	Follow up time
ansaNSR	Hassan 2012 [34]	9	HNR	7.60	8.10	24 m
	Lee 2018 [40]	19	HNR	17.10	21.8*	36 m
	Li 2014 [42]	349	NHR	0.18	0.02*	> 24 m
	Wang 2011 [56]	237	NHR	0.15	0.018*	> 24 m
	Wang 2011 [57]	56	NHR	0.14	0.026*	> 24 m
	Wang 2020 [58]	37	NHR	0.10	0.038*	12 m
	Wang 2020 [59]	13	NHR	0.07	0.06	24 m
	Ab Rani 2021 [25]	9	NHR	0.32	0.06	12 m
NMPR	Hassan 2011[33]	13	HNR	4.00	9.00*	> 24 m
	Hassan 2014 [32]	11	HNR	3.80	9.00*	> 24 m
	Kodama 2017 [36]	40	HNR	4.00	8.50*	> 12 m
	Sanuki 2015 [52]	12	HNR	4.90	8.60	24 m
	Yumoto 2010 [63]	22	HNR	5.54	8.02*	12–36 m
	Nishimoto 2023 [48]	68	HNR	4.20	7.90	24 m
	Kodama 2022 [37]	29	NHR	0.70	0.20	24 m
RRNSA	Yumoto 2006 [62]	1	HNR	NR	17.10	NR
RRNSANG	Yumoto 2006 [62]	8	HNR	NR	17.10	NR
	Li 2013 [43]	14	NHR	0.15	0.02*	24 m

*ansaNSR * ansa cervicalis non-selective reinnervation, *NMPR * nerve-muscle pedicle reinnervation, *RRNSA * recurrent to recurrent non-selective anastomosis, *RRNSANG * recurrent to recurrent non-selective anastomosis with interposition nerve graft, *NR * not reported,

*=reported as statistically significant improvement

**Table 7:** Aerodynamic analysis – Maximum phonation time

MPT	Study	Patients (*n*)	Preoperative mean(SD)	Postoperative mean (SD)	Follow up time
ansaNSR	Ab Rani 2021 [25]	9	11.58 (4.88)	15.29 (5.82)	12 m
	Hassan 2012 [34]	9	5.00	15.00*	24 m
	Kumai 2016 [39]	8	NR	20.70	12 m
	Lee 2018 [40]	19	6.69 (1.82)	10.79 (1,64)*	36 m
	Li 2014 [42]	349	5.70	16.56*	> 24 m
	Marie 2020 [45]	48	7.00	12.72*	36 m
	Miyauchi 2009 [47]	65	NR	20.90 male 18.80 female	12 m
	Paniello 2011 [50]	12	4.20	6.50	12 m
	Su 2007 [53]	10	7.00 (1.22)	16.00 (5.52)*	> 24 m
	Wang 2011[56]	237	6.20	17.22*	> 24 m
	Wang 2011[57]	56	6.40	15.97*	> 24 m
	Wang 2020[58]	37	9.50	17.82*	12 m
	Wang 2020 [59]	13	15.10	16.10	24 m
	Yoshioka 2016 [60]	345	NR	15.00	12 m
	Yuan 2020 [61]	8	NR	15.10	> 12 m
	Candelo 2023[28]	5	8.00	10.50	18 m
NMPR	Hassan 2011[33]	13	5.00	20.00*	> 24 m
	Hassan 2014 [32]	11	5.40 (2.1)	21.50 (7.0)*	> 24 m
	Kodama 2015 [38]	33	4.00	18.00*	24 m
	Kodama 2017 [36]	40	4.00	17.00*	> 12 m
	Sanuki 2015[52]	12	4.30	17.50*	24 m
	Tanaka 2004 [54]	9	NR	14.70	24 m
	Yumoto 2010 [63]	22	5.20 (2.1)	17.50 (8.8)*	12–36 m
	Kodama 2022 [37]	29	4.00	18.00*	24 m
	Nishimoto 2023 [48]	68	4.60 (2.5)	15.30 (7.6)*	24 m
RRNSA	Miyauchi 2009 [47]	7	NR	20.90 male, 18.80 female	12 m
	Yoshioka 2016 [60]	59	NR	15.00	12 m
	Yuan 2020 [61]	8	NR	14.90	> 12 m
	Yumoto 2006 [62]	1	NR	15.10	NR
HNSR	Paniello 2000[5]	9	3.00	> 12,00	12 m
RRNSANG	Kumai 2016 [39]	9	NR	23.40	12 m
	Li 2013[43]	14	5.71	20.10*	24 m
	Miyauchi 2009 [47]	14	NR	20.90 male, 18.80 female	12 m
	Yoshioka 2016 [60]	35	NR	14.00	12 m
	Yuan 2020 [61]	4	NR	10.00	NR
	Yumoto 2006 [62]	8	NR	15.10	NR

*ansaNSR * ansa cervicalis non-selective reinnervation, *NMPR * nerve-muscle pedicle reinnervation, *RRNSA * recurrent to recurrent non-selective anastomosis, *RRNSANG * recurrent to recurrent non-selective anastomosis with interposition nerve graft, *HNSR * Hypoglossal non-selective reinnervation, *NR * not reported,

*=reported as statistically significant improvement

### Aerodynamic analysis

In 26 studies MPT was reported (total patients, *n* = 1635: 1230 ansaNSR, 237 NMPR + AA, 75 RRNSA, 84 RRNSANG, 9 HNSR) [5, 25, 28, 32–34, 36, 38–40, 42, 43, 45, 47, 48, 50, 52–54, 56–63]. Preoperative MPT ranged from 3.0 to 15.1 s and improved to 6.5–21.5 s postoperatively, with improvements of 2.3–16.1 s. The weighted mean MPT improvement was 9.8 s for ansaNSR and 12.8 s for NMPR + AA (Table 8).

**Table 8:** Visual analysis – Videostroboscopy glottal gap

	Study	Patients (*n*)	Used scale	Glottal gap pre	Glottal gap post	Follow up time
ansaNSR	Chhetri 1999 [20]	10	1–5, 5 = no gap	2,6	4,5*	36 m
	Kumai 2016 [39]	8	0–4, 0 = no gap	NR	1,1	12 m
	Lee 2018 [40]	19	0–3, 3 = no gap	0,4	2,4*	36 m
	Li 2014 [42]	349	0–3, 0 = no gap	3,0	0*	> 24 m
	Zheng 1996 [64]	8	glottal opening in mm	2,3	0,8	> 12 m
NMPR	Kodama 2015 [38]	33	0–4, 0 = no gap	3,6	0,5*	24 m
	Kodama 2017 [36]	40	0–4, 0 = no gap	3,8	0,8*	24 m
	Sanuki 2015 [52]	12	0–4, 0 = no gap	3,4	0,3*	24 m
RRNSANG	Kumai 2016 [39]	9	0–4, 0 = no gap	NR	0,8	12 m

*ansaNSR * ansa cervicalis non-selective reinnervation, *NMPR * nerve-muscle pedicle reinnervation, *RRNSANG * recurrent to recurrent non-selective anastomosis with interposition nerve graft, *NR * not reported,

*=reported as statistically significant improvement

### Videostroboscopy

Improvement in videostroboscopic findings, including glottal closure, vocal fold position, mucosal wave, phase symmetry and regularity, was reported in nine studies. None of these studies described outcomes in a way that would allow for the estimation of the underlying structural changes important to reinnervation such as an increase in muscle bulk, improved tension or better position of the arytenoid. The outcomes of glottal closure are presented in Table 5. Notably, not all authors use the same cutoff values. All results show an improvement in closure [20, 36, 38–40, 42, 52, 64]. Several other studies report improvements in glottal videostroboscopy findings but do not present supporting data. Wang et al. noted significant improvements in these parameters across four ansaNSR studies, yet did not provide patient-level details [56–59]. Su et al. reported vocal fold position improvement to the median position in 6/10 patients but noted no change in 2/10 patients, the result for the remaining 2 patients is unclear [53]. Maronian et al. found improved vocal fold position in 3/7 patients, stable position in 2/7 and suggestive worsening in 2/7. They state that this may be due to poor observer ability to perform subjective judgment of vocal fold position or because the main aim of reinnervation is to restore bulk of the muscle rather than glottal closure [46]. Rohde et al. reported improvements in all videostroboscopic evaluations for 4 recurrent-to-recurrent reinnervation cases (Table 9) [51].

**Table 9:** Acoustic analysis – Jitter/Shimmer

VHI	Study	Type of VHI	Patients (*n*)	Preoperative mean(SD)	Postoperative mean (SD)	Follow up time
ansaNSR	Ab Rani 2021 [25]	VHI-10	9	18.6 (18.08)	1.6 (2.57)*	12 m
	Buyukatalay 2021 [27]	VHI-10	6	28.2	15.2*	> 6 m
	Lee 2018 [40]	VHI-30	19	84.8 (16.9)	8.0*	36 m
	Candelo 2023[28]	VHI-30	5	83.0	7.5*	18 m
NMPR	Kodama 2014 [38]	VHI-10	40	21.0	5.0*	12 m
	Buyukatalay 2021 [27]	VHI-10	4	31.8	14.0*	> 6 m
	Kodama 2022 [37]	VHI-10	29	24.5 (7.3)	9.5 (6.9)*	20,9 m
	Nishimoto 2023 [48]	VHI-10	68	23.3 (8.1)	8.7 (7.4)*	24 m
RRNSA	Rohde 2012 [51]	VHI-30	4	no value	41.0	> 8 m
RRNSANG	Rohde 2012 [51]	VHI-30	3	no value	47.6	> 5 m

*ansaNSR * ansa cervicalis non-selective reinnervation, *NMPR * nerve-muscle pedicle reinnervation, *RRNSA * recurrent to recurrent non-selective anastomosis, *RRNSANG * recurrent to recurrent non-selective anastomosis with interposition nerve graft, *NR* not reported

*=reported as statistically significant improvement

### Subjective analysis

Eight studies assessed VHI, with five using VHI-10 [25, 27, 37, 38, 48] (total patients, *n* = 156: 15 ansaNSR, 141 NMPR + AA) and three using VHI-30 [28, 48, 51] (total patients, *n* = 31: 24 ansaNSR, 4 RRNSA, 3 RRNSANG). VHI-10 scores improved by 13.0 to 17.0 points (weighted mean: −15.4 for ansaNSR and − 15.2 for NMPR + AA), while VHI-30 improved by 75.5 to 76.8 points (weighted mean: −76.5 for ansaNSR). All studies reported significant postoperative improvements (Table 5).

### Predictive factors

- Age: studies showed that younger patients (under 60 years) tended to achieve better outcomes, though significant improvement was also noted in older groups.
- Resting position vocal fold and etiology of paralysis: none of the studies assessed these parameters as a predictor for outcome of reinnervation.
- Duration of denervation: better voice outcomes were observed when surgeries were performed within two years of denervation, though successful cases with longer durations were also reported.
- Residual (re)innervation/LEMG: none of the studies assessed this parameter as a predictor for outcome of reinnervation.

### Surgical complications

Only five studies reported complications, including minor issues such as ecchymosis, hematomas and infections [26, 28, 56, 57, 63]. Major complications (tracheostomy) were reported in two studies [57, 63].

## Discussion

### General outcome of non-selective laryngeal reinnervation

This review highlights that non-selective laryngeal reinnervation is effective for UVFP, with studies demonstrating high rates of voice improvement, primarily based on data on the ansaNSR (*N* = 1391) and NMPR + AA (*N* = 278) techniques [5, 20, 25–64]. Although not all parameters normalize, on average, patients can expect a 2-point improvement of the Grade score of the GRBAS. For the VHI-10 and VHI-30 respectively a 15- versus 77-point improvement, for both techniques, qualifying as a near-normal voice. Evidence of active reinnervation is further supported by objective physiological data showing increased postoperative muscle activation on LEMG, with motor unit and polyphasic potentials in both ansaNSR and NMPR [44]. The timeline for recovery is long with first signs of improvement reported as early as 3.5 weeks post-NMPR [55]. Other recovery times cited are 83 days [61] and six weeks to seven months [46]. Additionally, several studies report that voice quality, acoustic measures and perceptual outcomes continue to improve gradually over time, with significant progress noted beyond 12 months. These findings collectively highlight that improvements in voice function may take more than a year to fully manifest. This delay is due to the extended timeline required for nerve regeneration, synaptic integration and muscular adaptation. This aligns with the biological processes involved in nerve recovery and emphasizes the importance of long-term follow-up to assess the efficacy of non-selective reinnervation procedures [30, 34, 36, 45, 65]. Notably, no cases of voice deterioration were reported across studies, although some improvements did not reach statistical significance. Lorenz et al. reported one failure out of 38 ansaNSRs, which was successfully salvaged by medialization thyroplasty [44]. Olson et al. identified one failure in 12 ansaNSRs, attributing it to the patient’s advanced age (77 years) [49]. Wang et al. cited four failures in 237 cases, caused by short recurrent laryngeal nerve (RLN) stumps, postoperative bleeding leading to nerve disconnection and denervation durations exceeding three years [56]. Su et al. reported two failures in 10 ansaNSRs, one due to cricoarytenoid fixation [53]. Several studies emphasize the utility of laryngeal framework surgery as a fallback for cases where reinnervation does not sufficiently improve voice outcomes, although judging from the data it is seldom performed [34, 36, 41, 50].

### Non-selective laryngeal reinnervation combined with other procedures

Several studies combined reinnervation with static procedures such as injection augmentation or laryngeal framework surgery. Candelo et al. combined ansaNSR with collagen injections, reporting voice improvement beyond six months, which they therefore attributed to successful reinnervation [28]. Similarly, Marie et al. used autologous fat injections in 48 ansaNSR cases, attributing improvements beyond 12 months to successful reinnervation rather than to the augmentation effects [45]. Lee et al. treated 20 out of 25 unilateral vocal fold paralysis (UVFP) patients with ansaNSR procedures using gelfoam, micronized alloderm, or collagen injections to mitigate dysphonia caused by recurrent laryngeal nerve transection during reinnervation [41]. It is unclear whether the other studies intended injections to provide immediate improvement or to prevent deterioration caused by severing of residual RLN innervation. The latter indication has mainly been described in studies investigating immediate reinnervation after thyroidectomy with RLN sacrifice, where patients initially had normal preoperative voices. To our knowledge, no data exist regarding the frequency of residual innervation loss immediately after non-selective reinnervation in patients with chronic denervation, leaving it unclear how often this occurs and which patients are most at risk. Overall, these findings suggest that temporary augmentation mitigates postoperative dysphonia, whether pre-existent or aggravated. Whether non-selective reinnervation with augmentation yields better long-term voice outcomes than reinnervation without augmentation remains unknown, as the current literature lacks direct comparative studies on this topic. Theoretically, additional injection may even enhance the reinnervation process based on findings by Dedry et al. who propose this enhancement occurs via proprioceptive feedback activation patients receiving early injections for UVFP [66]. At this stage, it is premature to conclude that there is a potential reinnervation-enhancing effect of injection augmentation. To the best of our knowledge, other studies investigating this topic have not been published.

The most frequent combination with LFS was that of NMPR + AA. Several Japanese groups have reported good results from this technique. The concept is based on manually ensuring the correct positioning of the vocal fold process in the phonatory position and relying on reinnervation mainly for bulking up the thyroarytenoid/lateral cricoarytenoid complex. Additional adduction may also result from reinnervation of the lateral cricoarytenoid muscle [36, 38, 48, 52, 63]. Anecdotally, issues have been raised around the ability of ansaNSR reinnervation to correct a malpositioned arytenoid causing a large glottic gap with obstructed compensation. In the opinion of many experienced surgeons this same adjustment is often achievable through ansaNSR (personal communication). Two studies examined the combination of ansaNSR with laryngeal framework surgery procedures. Chhetri et al. compared ansaNSR combined with AA to AA alone in 10 patients and found no significant differences, potentially due to preexisting synkinetic reinnervation. They recommend intraoperative electromyography to identify truly denervated vocal folds that could benefit from ansaNSR versus vocal folds with synkinetic reinnervation, which may not gain additional benefit from ansaNSR [20]. Havas et al. studied nine cases with different combinations of procedures, but the heterogeneity of the interventions prevented the evaluation of the isolated effects of reinnervation [35].

### Reinnervation versus laryngeal framework surgery

Four studies compared the outcomes of ansaNSR or NMPR + AA to LFS only, which is the other main technique for obtaining durable voice improvement in patients with UVFP. Rani et al. reported that ansaNSR resulted in significantly better voice outcomes compared to medialization thyroplasty, as measured by the VHI-10 and MPT [25]. On the other hand, Paniello et al. found no significant difference between the two techniques [50]. Hassan et al. compared 11 patients undergoing NMPR + AA to 11 undergoing MT + AA. Both groups showed significant voice improvement after three months. However, the NMPR + AA group continued improving until the two-year follow-up, while the MT + AA group did not, resulting in a better outcome for the NMPR + AA group in the long term. The authors concluded that restoring the dynamic, tensioning abilities of the thyroarytenoid muscle through NMPR is functionally superior to the static bulking achieved with MT [32]. Kodama concluded that both treatments yielded similar outcomes across parameters such as flow rate, glottal gap, vibration amplitude and acoustic measures. However, vocal fold vibration regularity and MPT were better in the NMPR group [36]. Large-scale, randomized studies stratifying patients by glottic gap size and shape are needed to draw definitive conclusions, although logistical challenges would make such a study difficult to implement. Additionally, the added value of AA in LFS would have to be addressed, as this topic is debated. Many surgeons feel that AA can significantly improve voice outcomes in selected patients with a malpositioned arytenoid [14]. It is therefore questionable whether ansaNSR should be compared to MT only or to MT + AA, at least in selected patients.

### Predictive factors

When treating unilateral vocal fold paralysis, predictive factors such as patient age, UVFP etiology, vocal fold position, duration of denervation and preoperative laryngeal electromyography findings should be considered. These factors may also introduce bias when comparing surgical outcomes.

### Age

Age has been studied as a factor in non-selective reinnervation outcomes, with younger patients generally showing better results due to the declining nerve regeneration capacity with age [48, 50, 67]. Li et al. divided 349 ansaNSR patients into four age groups: A (< 30 years), B (30–44 years), C (45–59 years) and D (≥ 60 years). Significant differences in glottal closure, overall grade, shimmer and noise-to-harmonics ratio (NHR) were observed only between the youngest and oldest groups (A vs. D, B vs. D, C vs. D: *P* < 0.05), leading to the conclusion that ansaNSR is less effective in patients aged ≥ 60 years [42]. Paniello et al. compared ansaNSR (*n* = 12) and MT (*n* = 12), finding no significant overall differences. However, ansaNSR patients aged < 52 years performed significantly better than those aged > 52 years at 12 months postoperative for perceptual ratings (perceptual ratings by untrained listeners (RUL) and GRBAS). This age effect was not observed in the MT group, prompting the recommendation to consider age in UVFP treatment planning [50]. No definite age threshold has been established in the studies reviewed for the current paper, and regenerative capacity is viewed as declining gradually, with potential limits suggested between 60 and 70 years [67]. Nishimoto et al. studied 68 patients undergoing NMPR + AA, divided into four age groups (< 50, 50–60, 60–70, > 70 years). Significant vocal function improvements were observed in all groups at 24 months, with no major differences, except for enhanced pitch range over time in the 60–70-year group. They concluded that NMPR + AA is effective across age groups [48]. However, it remains unclear whether AA was necessary or if reinnervation alone would have sufficed to correct arytenoid malposition, introducing potential bias that limits its relevance for assessing age-related reinnervation outcomes.

### Duration of denervation

Alongside age, the success of reinnervation is likely influenced by the duration of denervation. A shorter denervation period appears to improve outcomes, though no strict cutoff point has been established. The only study assessing the effect of duration on voice improvement was conducted by Li et al., who divided 349 ansaNSR patients into three groups based on denervation duration: A (6–12 months), B (12–24 months) and C (> 24 months). All patients showed significant improvement after surgery, although groups A and B showed better outcomes for overall grade, jitter, shimmer, NHR, MPT and postoperative LEMG activity than group C, suggesting outcomes are better when surgery is performed within two years of nerve injury [42]. Despite these trends, several studies in this review have demonstrated the success of non-selective reinnervation, both in older patients and in those with long denervation durations. Crumley suggested in 1991 that even limited axonal sprouting may prevent complete denervation atrophy in the posterior cricoarytenoid, thyroarytenoid, or lateral cricoarytenoid muscles. This could potentially explain successful reinnervation in long-term denervation cases [30]. These factors should be considered and discussed with patients when evaluating non-selective reinnervation approaches versus alternatives such as laryngeal framework surgery. Some patients with long-term denervation or older age may still prefer to undergo non-selective reinnervation, with LFS as a salvage option in case of inadequate results, although this is a time-consuming strategy. Especially, if a procedure under local anesthesia, which is mostly preferred for LFS, is not feasible.

### Laryngeal electromyography

LEMG is a valuable diagnostic tool for assessing nerve injury and predicting the chance of spontaneous recovery. It confirms nerve damage, detects early signs of reinnervation and aids in preoperative planning by assessing the state of denervation and reinnervation [68]. The timing of LEMG is important, as electrophysiological findings depend on the extent and duration of denervation [69]. LEMG findings are limited in the acute phase of neural damage because muscle fibers take time to become electrically unstable following denervation. This lag explains the absence of fibrillations or positive sharp waves early on. In cases of axonotmesis or neurotmesis, nerve fibers begin degenerating, but this process takes several days to fully affect the distal muscle. Therefore, most authors recommend performing LEMG within 2–6 weeks of onset for optimal evaluation. In this early phase, signs of spontaneous muscle activity, like fibrillation potentials and positive sharp waves will confirm nerve damage and low or absent motor unit activity can signal severe damage with poor recovery potential. Repeating LEMG in the intermediate phase (6–12 weeks) allows further assessment of the likelihood of recovery, as polyphasic motor unit potentials appear when regenerating axons begin to reinnervate denervated muscle fibers. Persistent spontaneous activity and absent motor unit recruitment at this stage strongly suggest a poorer prognosis. Prompt LEMG assessment therefore helps in the effective management of UVFP [46, 68, 69]. Marshall et al. correlated postoperative LEMG with standard evaluations, showing its utility in monitoring non selective reinnervation surgery outcomes [70]. Maronian et al. used LEMG to exclude patients with partial reinnervation, arguing that spontaneous recovery was likely in these cases [46]. However, the role of LEMG in preoperative decision-making remains unclear, as studies often do not specify how denervation patterns influenced treatment timing or patient counseling [46, 50, 52, 59, 67]. Also, LEMG was only performed in a quarter of the studies reviewed, meaning the data are based on a minority of the papers. Anecdotally, it has also been discussed as a tool to select patients suitable for non-selective reinnervation, although views on the interpretation of findings vary. Some prefer to see some motor unit activity as this signals a muscle that will more actively promote and accept new innervation whereas others argue that no activity is preferable as the larynx will respond better to reinnervation from single rather than multiple sources (personal communications). To the best of our knowledge there is no data furthering this discussion.

Finally, as none of the studies evaluated outcomes based on etiology or vocal fold position (i.e. malposition of the arytenoid), no conclusions could be drawn as to the impact of these potentially predictive factors on reinnervation success. Preoperative laryngeal electromyography (LEMG) was reported in only a quarter of our included studies, highlighting the need for further prospective research to clarify its clinical utility.

### Previous reviews

Our findings align with prior systematic reviews on laryngeal reinnervation. Aynehchi et al. analyzed 14 studies (1966–2009, *N* = 329), all reporting improved outcomes post-surgery [9]. Marshall et al. reviewed 11 studies (up to April 2022, *N* = 440) and found that LEMG data were consistent with perceptual, acoustic and visual analyses, demonstrating its reliability in assessing surgical success [70]. Fadhil evaluated 28 studies (2001–2022, *N* = 2032) to determine the optimal timing for ansaNSR. They suggested earlier repairs may yield better outcomes but could not establish a definitive timing threshold [65]. Onifade et al. reviewed 27 studies (up to October 2022, *N* = 803, including 747 non-selective cases), concluding that non-selective laryngeal reinnervation improves voice outcomes. A meta-analysis of maximum phonation time (MPT) showed a statistically significant increase of 1.32 s (CI 0.79–1.85), although the effect size may be clinically modest [71].

These reviews collectively demonstrate the effectiveness of laryngeal reinnervation as a treatment option for UVFP. However, it would be valuable to see these positive results confirmed in a prospective trial based on a comprehensive voice analysis protocol. Notably, only a fifth of studies included valuable clinical parameters such as the VHI (8 out of 42) [25, 27, 28, 37, 38, 40, 48, 51] and perceptual evaluation (18 out of 42) [28, 29, 32–34, 36–38, 42, 43, 45, 48, 51, 52, 56–59]. Additionally, a prospective study would allow for pre-determined definitions of insufficient results or failures and would permit monitoring for temporary aggravation of dysphonia. Additional challenges that persist include determining the feasibility of double reinnervation (i.e. from residual recurrent activity and NMPR of the thyroarytenoid muscle), the potential correction of arytenoid position and the predictive value of LEMG findings for non-selective reinnervation surgery success. Currently, in lieu of evidence from large series, a clinical consensus and/or best practice statement addressing these topics could be a first step to advance clinical decision-making and identify the priorities for further investigation.

### Limitations

This review has several limitations. First, the studies included are retrospective, leading to both within- and between-study heterogeneity in patient populations, follow-up duration and voice outcome parameters employed. This inherently introduces bias that is difficult to control. Notably, most studies followed patients for at least one year which is important for reliably establishing the outcomes of these procedures. This heterogeneity is the reason we did not find the data suitable for meta-analysis. However, we did calculate weighted average outcomes for the most relevant clinical parameters to give some sense of what can be expected from these procedures. These values must therefore be interpreted as indicative trends. Standardized voice evaluation methods, as proposed by Desuter et al., could improve study consistency [72]. Similarly, defined classifications of glottic defects and standardized LEMG reporting would also help identify patient subcategories with better outcomes.

The data available in this review was mainly on ansaNSR and NMPR + AA, putting a focus on these two techniques in the discussion. This does not rule out good outcomes with other reinnervation techniques, but their sample sizes were too small for meaningful analysis.

Referring back to Fig. 1, we found that while several studies published data on stroboscopy, there was no information on the actual structural changes that are fundamental to the success of reinnervation: increase in muscle bulk, improvement of tension and improvement in the position of the arytenoid. We grant that these parameters are difficult to measure or estimate but would recommend that future studies generate data on these aspects. Equally, it is vital that studies employ more uniform voice outcome parameters and timing of evaluation. While this review provides a comprehensive summary of existing literature up until 15 October 2023, new series are regularly being published to supplement the knowledge in this area. To the best of our knowledge, however, more recent data does not necessitate significant adjustment of our findings. Finally, although this is a review of scientific data, its value lies mainly in guiding clinicians in counseling patients on reinnervation options and identifying the gaps in the literature relevant to those who make treatment choices between modalities in daily practice.

## Conclusion

Non-selective laryngeal reinnervation, particularly ansaNSR and NMPR + AA, is a safe and effective technique for many UVFP patients. Despite data heterogeneity precluding meta-analysis, most studies report significant improvements, with few patients experiencing no benefit. The timespan for improvement is broad and typically ranges from several months to over a year. Determining whether non-selective laryngeal reinnervation is superior to other techniques remains challenging due to data heterogeneity. Each UVFP case requires thorough multidisciplinary evaluation, factoring in patient age and denervation duration, which currently appear to be the most influential predictive factors. While no strict cut-off exists, thresholds of 60 years of age and 2 years of denervation are suggested, though successful outcomes beyond these limits are frequently reported. In lieu of much-needed further studies, a consensus statement addressing key uncertainties—such as the impact of non-selective reinnervation techniques on arytenoid position, the efficacy of dual innervation, the predictive value of the preoperative LEMG and the recommendations on possible age and denervation duration limits—would be of great value.

## Supplementary information

Below is the link to the electronic supplementary material.

Supplementary material 1 (DOCX 13.4 KB)

Supplementary material 2 (DOCX 62.7 KB)

## Abbreviations

AA: Arytenoid adduction
ANSA-NSR: Ansa cervicalis non-selective reinnervation
GRBAS: Grade Roughness Breathiness Asthenia Strain
HNR: Harmonics-to-noise ratio
HNSR: Hypoglossal non-selective reinnervation
LEMG: Laryngeal electro myography
LFS: Laryngeal framework surgery
MINORS: Methodological Index for Non-Randomized Studies
MPT: Maximum phonation time
MT: Medialisation thyroplasty
NHR: Noise-to-harmonics ratio
NMPR: Nerve muscle pedicle reinnervation
PRISMA: Preferred Reporting Items for Systematic Reviews and Meta-Analyses
PROSPERO: Prospective Register of Systematic Reviews
RRNSA: Recurrent-to-recurrent non-selective anastomosis
RRNSANG: Recurrent-to-recurrent non-selective anastomosis with interposition nerve graft
UVFP: Unilateral vocal fold paralysis
VHI: Voice Handicap Index
V-RQOL: Voice-related quality of life
