# Supplementary material for: Laryngeal reinnervation for unilateral vocal fold paralysis in adults; a systematic review of the literature for the clinician
Source: Eur Arch Otorhinolaryngol. 2025 Oct 15;282(11):5779–95. doi: 10.1007/s00405-025-09737-7 (PMC12605593; doi:10.1007/s00405-025-09737-7)
Supplement: Supplementary file 1 — Supplementary material 1 (DOCX 13.4 KB) [file 405_2025_9737_MOESM1_ESM.docx]

**Supplementary material 1: Search strategy**

((("recurrent laryngeal nerve"[mesh] OR "recurrent laryngeal nerve"[tw] OR "recurrent laryngeal nerves"[tw]) AND ("Unilateral vocal fold paralysis"[tw] OR "Unilateral vocal fold paresis"[tw] OR "Unilateral vocal fold palsy"[tw] OR "Unilateral vocal paralysis"[tw] OR "Unilateral vocal paresis"[tw] OR "Unilateral vocal palsy"[tw] OR "Unilateral vocal folds paralysis"[tw] OR "Unilateral vocal folds paresis"[tw] OR "Unilateral vocal folds palsy"[tw] OR (("Vocal Cord Paralysis"[mesh] OR "Laryngeal Nerve Palsy"[tw] OR "Laryngeal Paralysis"[tw] OR "Vocal Cord Palsy"[tw] OR "Vocal Cord Paralysis "[tw] OR "Vocal Cord Paresis"[tw] OR "Vocal Fold Palsies"[tw] OR "Vocal Fold Palsy"[tw] OR "Vocal Fold Paralysis"[tw] OR ((" Vocal Cords"[mesh] OR "Vocal Cord"[tw] OR "Vocal Cords"[tw] OR "Vocal Fold"[tw] OR "Vocal Folds"[tw]) AND ("Palsy"[tw] OR "Paralysis "[tw] OR "Paresis"[tw]))) AND ("Unilateral"[tw] OR "Unilateral*"[tw] OR "Uni lateral"[tw] OR "uni and bilateral"[tw]))) AND ("surgery"[subheading] OR "surgery"[tw] OR "surgical*"[tw] OR "Surgical Procedures, Operative"[Mesh]) NOT ("Animals"[mesh] NOT "humans"[mesh]) AND English[la]) OR ("Recurrent Laryngeal Nerve/surgery"[majr] NOT ("bilateral*"[ti] NOT "unilateral*"[ti]) NOT ("Animals"[mesh] NOT "humans"[mesh]) AND English[la]) OR ("Vocal Cord Paralysis/surgery"[mesh] NOT ("bilateral*"[ti] NOT "unilateral*"[ti]) NOT ("Animals"[mesh] NOT "humans"[mesh]) AND English[la]) OR (("Unilateral vocal fold paralysis"[tw] OR "Unilateral vocal fold paresis"[tw] OR "Unilateral vocal fold palsy"[tw] OR "Unilateral vocal paralysis"[tw] OR "Unilateral vocal paresis"[tw] OR "Unilateral vocal palsy"[tw] OR "Unilateral vocal folds paralysis"[tw] OR "Unilateral vocal folds paresis"[tw] OR "Unilateral vocal folds palsy"[tw] OR (("Vocal Cord Paralysis"[mesh] OR "Laryngeal Nerve Palsy"[tw] OR "Laryngeal Paralysis"[tw] OR "Vocal Cord Palsy"[tw] OR "Vocal Cord Paralysis "[tw] OR "Vocal Cord Paresis"[tw] OR "Vocal Fold Palsies"[tw] OR "Vocal Fold Palsy"[tw] OR "Vocal Fold Paralysis"[tw] OR ((" Vocal Cords"[mesh] OR "Vocal Cord"[tw] OR "Vocal Cords"[tw] OR "Vocal Fold"[tw] OR "Vocal Folds"[tw]) AND ("Palsy"[tw] OR "Paralysis "[tw] OR "Paresis"[tw]))) AND ("Unilateral"[tw] OR "Unilateral*"[tw] OR "Uni lateral"[tw] OR "uni and bilateral"[tw]))) AND ("laryngeal reinnervation"[tw] OR "laryngeal re innervation"[tw] OR "larynx reinnervation"[tw] OR "laryngeal nerve reinnervation"[tw] OR (("Laryngeal Nerves"[Mesh] OR "Larynx"[mesh] OR "Laryngeal Diseases"[Mesh] OR "laryn*"[tw]) AND ("re innerv*"[tw] OR "reinnerv*"[tw] OR "Nerve Regeneration"[Mesh]))) AND ("Voice Quality"[Mesh] OR "Voice Quality"[tw] OR "Voice Qualities"[tw] OR "Vocal Quality"[tw] OR "Vocal Qualities"[tw] OR "quality of voice"[tw] OR "Hoarseness"[Mesh] OR "Hoarseness"[tw] OR "Hoars*"[tw] OR "Phonation"[Mesh] OR "Phonation"[tw] OR "vocal sound"[tw] OR "vocal sounds"[tw] OR "Speech Quality"[tw] OR "Speech Qualities"[tw] OR "Sound Quality"[tw] OR "Sound Qualities"[tw] OR "voice outcomes"[tw] OR "voice outcome"[tw] OR "voice results"[tw] OR "voice result"[tw] OR "voice analysis"[tw] OR "voice improvement"[tw] OR "voice deterioration"[tw] OR (("Voice"[mesh] OR "voice"[tw]) AND ("improvement"[tw] OR "deterioration"[tw] OR "improv*"[tw] OR "deteriorat*"[tw] OR "quality"[tw] OR "qualities"[tw]))) NOT ("Animals"[mesh] NOT "humans"[mesh]) AND English[la]))
