# Supplementary material for: Laryngeal reinnervation for unilateral vocal fold paralysis in adults; a systematic review of the literature for the clinician
Source: Eur Arch Otorhinolaryngol. 2025 Oct 15;282(11):5779–95. doi: 10.1007/s00405-025-09737-7 (PMC12605593; doi:10.1007/s00405-025-09737-7)
Supplement: Supplementary file 2 — Supplementary material 2 (DOCX 62.7 KB) [file 405_2025_9737_MOESM2_ESM.docx]

**Supplementary material 2:** Included studies

| Author | Year of publication | Non selective reinnervation technique(s) | Total patients | Risk of Bias score |
| --- | --- | --- | --- | --- |
| Ab Rani [25] | 2021 | ansaNSR | 9 | 11 |
| Blumin [26] | 2008 | RRNSA | 16 | 11 |
| Buyukatalay [27] | 2021 | ansaNSR n=6, NMPR n=4 | 10 | 10 |
| Candelo [28] | 2023 | ansaNSR | 5 | 9 |
| Chhetri [20] | 1999 | ansaNSR + AA | 10 | 7 |
| Chou [29] | 2003 | RRNSA | 8 | 8 |
| Crumley [30] | 1991 | ansaNSR | 12 | 7 |
| Dzodic [31] | 2016 | ansaNSR n=14, RRNSA n=7 | 21 | 8 |
| Hassan [33] | 2011 | NMPR +AA | 13 | 10 |
| Hassan [34] | 2012 | ansaNSR + AA | 9 | 10 |
| Hassan [32] | 2014 | NMPR +AA | 11 | 10 |
| Havas [35] | 1999 | RRNSA n=3, RRNSANG n=3, NMPR n=6 | 12 | 5 |
| Kodama [38] | 2015 | NMPR +AA | 33 | 11 |
| Kodama [36] | 2017 | NMPR +AA | 40 | 9 |
| Kodama [37] | 2024 | NMPR +AA n= 28, NMPR n=1, ansaNSR n=1 | 30 | 10 |
| Kumai [39] | 2016 | ansaNSR n=8, RRNSANG, n=9 | 17 | 8 |
| Lee [40] | 2018 | ansaNSR | 19 | 10 |
| Lee [41] | 2007 | ansaNSR | 25 | 8 |
| Li [43] | 2013 | RRNSANG | 14 | 10 |
| Li [42] | 2014 | ansaNSR | 349 | 10 |
| Lorenz [44] | 2008 | ansaNSR | 46 | 10 |
| Marie [45] | 2020 | ansaNSR | 48 | 12 |
| Maronian [46] | 2003 | NMPR n=3, ansaNSR n=6 | 9 | 9 |
| Miyauchi [47] | 2009 | RRNSA n=7, RRNSANG n=14, ansaNSR n=65 | 86 | 5 |
| Nishimoto [48] | 2023 | NMPR +AA | 68 | 9 |
| Olson [49] | 1998 | ansaNSR | 12 | 8 |
| Paniello [5] | 2000 | HNSR | 9 | 7 |
| Paniello [50] | 2011 | ansaNSR | 12 | 13 |
| Rohde [51] | 2012 | RRNSA n=4 , RRNSANG n=3, SVR n=2 | 9 | 12 |
| Sanuki [52] | 2015 | NMPR +AA | 12 | 8 |
| Su [53] | 2007 | ansaNSR | 10 | 14 |
| Tanaka [54] | 2004 | NMPR | 9 | 9 |
| Tucker [55] | 1981 | NMPR +AA | 27 | 5 |
| Wang [56] | 2011 | ansaNSR | 237 | 11 |
| Wang [57] | 2011 | ansaNSR | 56 | 9 |
| Wang [58] | 2020 | ansaNSR | 37 | 13 |
| Wang [59] | 2020 | ansaNSR | 13 | 8 |
| Yoshioka [60] | 2016 | RRNSA n=59, ansaNSR n=345 ,RRNSANG n=35 | 439 | 4 |
| Yuan [61] | 2020 | RRNSA n=8, ansaNSR n=8 , RRNSANG n=4 | 20 | 10 |
| Yumoto [62] | 2006 | RRNSA n=1, RRNSANG n=8 | 9 | 10 |
| Yumoto [63] | 2010 | NMPR +AA | 22 | 10 |
| Zheng [64] | 1996 | ansaNSR | 8 | 9 |

ansaNSR = ansa cervicalis non-selective reinnervation, NMPR = nerve-muscle pedicle reinnervation, RRNSA = recurrent to recurrent non-selective anastomosis, RRNSANG = recurrent to recurrent non-selective anastomosis with interposition nerve graft, HNSR = Hypoglossal non-selective reinnervation, AA = arytenoid adduction
